# Characterization of the Flavor Precursors and Flavor Fingerprints in Grazing Lambs by Foodomics

**DOI:** 10.3390/foods11020191

**Published:** 2022-01-12

**Authors:** Yuanyuan Yang, Jing Li, Xueting Jia, Qingyu Zhao, Qing Ma, Yanan Yu, Chaohua Tang, Junmin Zhang

**Affiliations:** 1 State Key Laboratory of Animal Nutrition, Institute of Animal Sciences of Chinese Academy of Agricultural Sciences, Beijing 100193, China; yangyuan2018@163.com (Y.Y.); CHNlijing@outlook.com (J.L.); jiaxueting2018@163.com (X.J.); zhaoqingyu@caas.cn (Q.Z.); yuyanan@caas.cn (Y.Y.); tangchaohua@caas.cn (C.T.); 2 Scientific Observing and Experiment Station of Animal Genetic Resources and Nutrition in North China of Ministry of Agriculture and Rural Affairs, Institute of Animal Sciences of Chinese Academy of Agricultural Sciences, Beijing 100193, China; 3 Institute of Animal Science, Ningxia Academy of Agricultural and Forestry Sciences, Yinchuan 750002, China; maqing1973@126.com

**Keywords:** feeding system, fatty acid, hydrophilic metabolite, volatile compound, Tan sheep

## Abstract

Tan sheep are greatly preferred by consumers in China because of their nutritional value and unique flavor. However, the meat quality of Tan sheep has decreased due to the change in feeding systems from grazing to indoor. Studies investigating the mechanisms for the decrease in meat quality are limited. A total of 28 Tan sheep were randomly allocated to two treatments, receiving a concentrated diet, or pasture. Flavor precursors and volatile compounds were analyzed with foodomics. E-nose and E-tongue analyses suggested that the aroma and taste profiles differed between the feeding systems. The grazing lambs had higher levels of linoleic acid and *n*-3 polyunsaturated fatty acids (*p* < 0.01). Metabolomics indicated that 25 hydrophilic metabolites active in glycolipid and amino acid metabolisms were changed by the feeding system. Among the 57 volatile compounds identified, methional, γ-butyrolactone, benzaldehyde, and ethyl acetate were at concentrations significantly higher in the grazing lambs than the indoor-fed lambs (*p* < 0.01). These results reveal key changes in flavor precursors and flavor profiles affected by the feeding system, which may provide an initial view of the reason for consumer preference for the grazing Tan sheep.

## 1. Introduction

Tan sheep, one of the domestic ovine breeds, are famous for their nutritional value and delicious flavor, and are highly preferred by consumers in China [1]. After decades of grazing restrictions, Tan sheep have been fed mainly a concentrated diet rather than fed with natural pasture. This results in a decrease in meat quality, reducing consumer satisfaction. Meat flavor is a strong driver of consumer liking for lamb [2]. Hence, understanding the underlying reason for the unique flavor of the grazing Tan sheep is necessary to improve indoor-fed lamb quality, and meet consumer demand.

Meat flavor is decided largely by some volatile compounds, which are mainly generated from lipids and water-soluble compounds by means of the Maillard reaction and lipid oxidation upon processing [3,4]. The lipid-generated components are mostly accountable for the aroma differences between species [5]. Many carbonyl compounds with low thresholds originate from the degradation of unsaturated fatty acids and greatly contribute to flavor formation [6,7]. Furthermore, polyunsaturated fatty acids can influence the formation of Maillard reaction products, thereby altering meat flavor [8]. On the other hand, water-soluble compounds strongly contribute to the formation of a basic meaty odor [9]. It is reported that methionine and cysteine are regarded as the major donors to the formation of meat flavor. Methionine could be degraded to methional, which has been considered as an important contributor to lamb aroma [6,10]. Therefore, determining the flavor precursors in detail is beneficial to understanding the flavor of grazing lambs.

According to previous studies, the feeding regime highly impacted the fatty acid composition, volatile profiles, and sensory quality [11,12,13]. Wang and his co-workers demonstrated that amino acid and fatty acid compositions were regulated under the grazing system by reprogramming the rumen bacterial community, thus enhancing meat quality [14]. Besides, they found that the grazing system modified the fatty acid content through rescheduling the lipid metabolites and the expression levels of lipid-metabolism-related genes, such as fatty acid binding protein 3, phospholipase A2 receptor, and low-density lipoprotein receptor [15]. Research regarding flavor fingerprints has mostly attended to the feeding regimes’ influence on volatile compounds [11,12,16] and specific-plant-derived compounds, namely terpenoids, phytol, and indoles [17,18,19]. However, few works have analyzed volatile profiles of Tan sheep under natural pasture and indoor feeding conditions [20] in detail, especially systemically investigating the flavor precursors and volatile compounds. Therefore, revealing the flavor attributes from the perspective of the precursor and volatile compounds is more conducive to understanding the possible reason for the unique flavor of the grazing lamb.

The purpose of this study was to (1) identify with E-nose and E-tongue analysis the taste and aroma profiles of Tan sheep fed with a concentrated diet or natural pasture; (2) characterize the flavor precursor and volatile compound profiles of the graze-fed and indoor-fed lambs; (3) unveil the key alterations of flavor attributes in lamb under different feeding conditions. The results may provide a basis for improving the flavor traits of indoor-fed Tan sheep.

## 2. Materials and Methods

### 2.1. Animal and Sample Collection

All procedures employed were approved by the Animal Care and Use Committee of the Institute of Animal Sciences of the Chinese Academy of Agricultural Sciences with permission number IAS 2019-76. In total, 28 male Tan sheep with an average body weight of 28.4 ± 2.5 kg, aged 4 months old, were randomly assigned into two groups: the concentrate-fed group (*n* = 14, CF, twice a day at 07:00 and 18:00) and pasture-fed group (*n* = 14, PF, from 16:00 to 02:00). The CF group were fed a total mixed ration; the ingredients of which are listed in Table S1. The PF group grazed on the dominant grass species of *Licorice*, *Sophora alopecuroides*, *Codonopsis pilosula*, *Astragalus*, *Alfalfa*, and *Caragana*. The experiment lasted 60 days. Following a 24 h fast, animals were electrically stunned, bled, skinned, and eviscerated in a commercial abattoir. At 45 min postmortem, longissimus thoracis samples were excised from each left and right carcass. After removing any visible external fat and connective tissue, each longissimus thoracis from the right carcass between the twelfth and thirteenth rib was cut into small pieces and frozen at −80 °C for further analysis. After determining muscle pH and meat color parameters at 45 min postmortem, the remaining samples were vacuum-packed and chilled (2–4 °C) for 24 h to measure meat quality and flavor traits.

### 2.2. Analysis of Meat Quality Attributes and Intramuscular Fat (IMF) Content

Muscle pH of the left longissimus thoracis at the seventh and eighth thoracic vertebra was measured in triplicates at 45 min (pH_45 min_) and 24 h (pH_24 h_) after slaughter, using an HI99163 acidimeter (Hanna Instruments Inc., Washington, RI, USA), which was calibrated by using pH 4.0 and pH 7.0 calibration solutions prior to measurement. Meat color parameters of the left longissimus thoracis at the eighth rib were determined at 45 min (L*_45 min_, a*_45 min_, and b*_45 min_) and 24 h (L*_24 h_, a*_24 h_, and b*_24 h_) after the slaughter on the surface of the cross-section of samples, after 45 min of blooming through a tintometer (CR-400, Konica Minolta Sensing Inc., Osaka, Japan). Prior to determination, this tintometer was corrected against a standard plate (2° observer angle, 8 mm aperture, D65 illuminant). Drip loss of the left longissimus thoracis was calculated as the percentage of the weight variance before and after storing, as previously reported [21]. Briefly, the rectangle-shaped samples (2 cm × 2 cm × 3 cm) were excised at the eighth and ninth thoracic vertebra at 24 h postmortem, initially weighed, suspended along the muscle-fiber direction in a plastic bag at 4 °C for 24 h, and reweighed. For determination of cooking loss, samples from the left longissimus thoracis between the ninth and thirteenth rib were cooked at 80 °C until the core temperature reached 70 °C. The weight loss before and after cooking dividing by the initial weight was defined as cooking loss. After taking cooking loss measurements, the cooked samples were subjected to measuring by shear force and texture profile analysis [21]. On analysis of shear force, six rectangle-shaped samples along the fiber direction were analyzed with a texture analyzer equipped with a HDP/BSW Warner–Bratzler blade (TA.XT.Plus, Stable Micro Systems, Godalming, Surrey, UK). Furthermore, six square-shaped samples with a cross-section of 1 cm^2^ were extracted. The texture profile parameters were analyzed using a TA.XT.Plus texture analyzer (Stable Micro Systems) equipped with a 75-mm-diameter cylinder probe. The probe moved down at a speed of 2.0 mm/s (pre-test), 1.0 mm/s (test), and 1.0 mm/s (post-test). The test parameters consisted of a trigger force of 5.0 g, a crosshead speed of 1 mm/s, a compression of 75%, and a time interval between two compressions of 5.0 s. Soxhlet extraction was used to determine IMF content according to the previous report [21]. The IMF content was defined as the percentage of the lipid weight in 100 g of wet muscle tissue.

### 2.3. E-Nose Analysis

Aroma profile analysis was performed by employing the PEN3 E-nose (Winmuster Airsense Analytic Inc., Schwerin, MV, Germany) with ten semiconductors and the sensors listed in Table S2. In brief, each sample (1.0 g) was added to a 10 mL airtight vial, incubated for 20 min at 80 °C, equilibrated at room temperature for 30 min, and subsequently injected into the sensory array at a speed of 600 mL/min. The detection and cleaning procedures were carried out for 60 s and 300 s, respectively.

### 2.4. E-Tongue Analysis

Taste attributes including umami, saltiness, sourness, bitterness, astringency, and sweetness, were analyzed with the SA402B E-tongue (Intelligent Sensor Technology, Inc., Kanagawa, Japan). Prior to analysis, samples were mixed with ultrapure water (minced meat/ultrapure water ratio of 1:4, *m*/*v*), vortexed, centrifuged for 10 min at 10,000× *g*, and filtrated to collect supernatants. The extracts were then transferred into special beakers for E-tongue determination. The reference solution consisted of 0.3 mM tartaric acid and 30 mM potassium chloride. It was used as a reference with the taste information set to zero. Data collected by E-tongue were corrected with the reference solution and were analyzed.

### 2.5. Analysis of Fatty Acids

Fatty acid methyl esters were obtained through transesterification with acetyl chloride in methanol [22], injected with high-purity nitrogen as a carrier gas at a flow rate of 3.0 mL/min, and separated on a capillary column (DB-23, 0.25 mm × 60 m) connected to an HP 6890 gas chromatograph (Agilent Technologies, Santa Clara, CA, USA). The oven temperature program rose to 220 °C at a rate of 4 °C/min, adjusted to 250 °C at a rate of 5 °C/min, and was held at 250 °C for 5 min. The identification was conducted by comparison with authentic standards (Supelco 37-Component FAME Mix; Sigma-Aldrich, St. Louis, MO, USA). Fatty acids were quantified with methyl undecanoate as an internal standard, and expressed as the percentage of individual fatty acid in total fatty acids.

### 2.6. Metabolomic Analysis

Metabolomic analysis was performed, as previously reported [10]. In brief, samples (70 mg) were homogenized in 700 μL of prechilled HPLC-grade methanol/water mixture (*v*/*v* = 4:1), incubated, and centrifuged at 20,000× *g* for 20 min. The supernatant was collected and concentrated to dryness.

The metabolites were analyzed on a TSQ Quantiva (Thermo Fisher Scientific, Waltham, MA, USA) equipped with a column of Atlantis HILIC (2.1 mm × 100 mm) and a column of BEH Amide (2.1 mm × 100 mm, Waters, Milford, MA, USA) in the positive and negative ion mode, respectively. Mobile phase A consisted of 15 mM acetate and 10 mM tributylamine in water. Mobile phase B was 15 mM acetate containing 10 mM tributylamine in 100% methanol. The gradient used was from 5% B to 90% B in 25 min. Mass spectrometer parameters were set as follows: a capillary temperature of 320 °C, a spray voltage of 3000 V, a source voltage of 3500 V in the positive ion mode, and 2500 V in the negative ion mode, and a sheath gas flow rate of 35 Arb. Metabolites were identified with TraceFinder 3.2 (Thermo Fisher Scientific).

### 2.7. Volatile Compound Analysis

After thawing, 2 g finely chopped samples were added to the vials in triplicate, incubated for 20 min at 80 °C, and subsequently injected into FlavourSpec^®^ gas chromatography–ion mobility spectrometry (GC–IMS, G.A.S. Instrument, Dortmund, Germany). Volatile compounds were separated on a column of FS-SE-54-CB-1 (15 m × 0.53 mm; CS-Chromatographie Service GmbH, Langerwehe, Germany) kept at 60 °C. High-purity nitrogen was used as the carrier gas with the programmed flow: 2 mL/min (2 min), 15 mL/min (8 min), 100 mL/min (10 min), and 150 mL/min (5 min). What eluted from the column was ionized and delivered to the drift tube using nitrogen at a 150 mL/min flow rate. Volatile compounds were identified by comparison with authentic standards in the GC–IMS library (G.A.S. Instrument).

### 2.8. Datal Analysis

The data were presented as the mean ± standard deviation. *T*-tests were carried out by using SPSS software (Version 23.0, SPSS Inc., Chicago, IL, USA) and *p* < 0.05 was used to represent statistical significance. We screened significantly different metabolites by univariate statistical analysis, and the criteria for significantly different metabolites were false discovery rate < 0.05 based on *T*-tests. Principal component analysis (PCA), heat map visualization, and pathway analysis were carried out with MetaboAnalyst (Version 5.0, www.metaboanalyst.ca, accessed on 16 August 2021). Bar charts were created using GraphPad Prism (Version 8.0, GraphPad Software Inc., La Jolla, CA, USA).

## 3. Results

### 3.1. Meat Quality Characteristics

As displayed in Table 1, the PF group had significantly higher pH_45 min_, pH_24 h_, a*_45 min_, L*_24 h_, and a*_24 h_ values relative to the CF group (*p* < 0.05). The PF samples showed remarkably higher shear force values compared with the CF samples (*p* < 0.01). Regarding texture profile, significant differences were observed for hardness, cohesiveness, and chewiness (*p* < 0.05). By contrast, IMF content significantly decreased in the PF group against the CF group (*p* < 0.01).

### 3.2. E-Nose and E-Tongue Analysis

**Figure 1:**
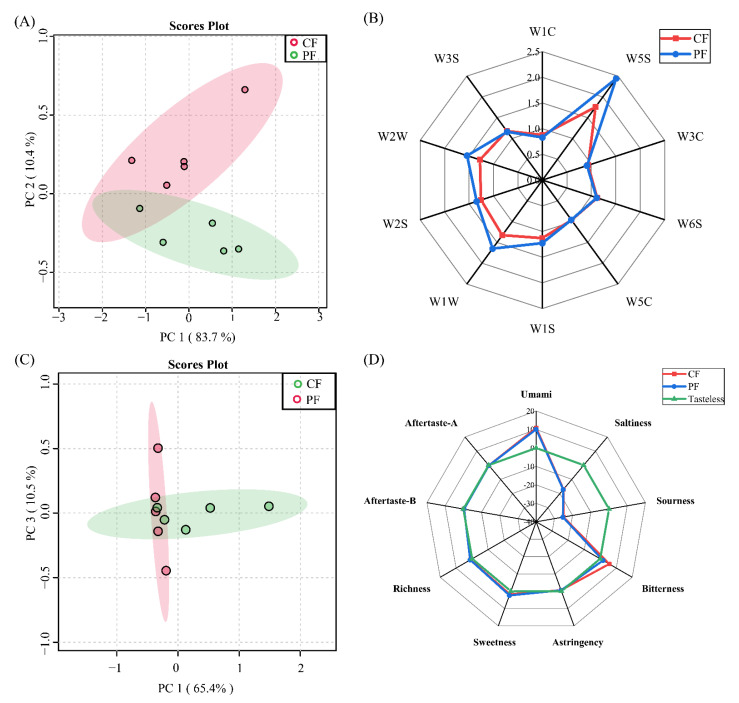
Aroma and taste profiles in concentrate-fed (CF) and pasture-fed (PF) groups (*n* = 5). Principal component analysis score plots and radar charts of the E-nose response values (**A**,**B**) and the E-tongue taste values (**C**,**D**).

To initially assess differences in the aroma and taste profiles of the CF and PF groups, the aroma and taste profiles were established by performing E-nose and E-tongue analyses. According to the PCA results, the first two principal components explained 94.1% of the variance in aroma, and samples from the CF and PF groups were separated from each other (Figure 1A). This result indicates that the overall aroma profile was different between the CF and PF groups. The response values of sensors including W5S, W1C, W2W, W2S, W1W, W6S, W3C, W1S, and W3S differed between the CF and PF groups (Figure 1B), indicating that the feeding system had an influence on aldehydes, alcohols, *N*- or S-containing compounds. Additionally, as shown in Figure 1C, principal components explained 75.9% of the total variance in taste profiles, with principal component 1 and principal component 3 representing 65.4% and 10.5%, respectively, reflecting the taste characteristics of lambs under different feeding systems. The PF samples tended to be separated from the CF samples, suggesting that the taste profile differed between the grazing and indoor-fed lambs. The taste values of bitterness, umami, richness, and sweetness were above the tasteless point, and the bitterness values significantly reduced in the PF group against in the CF group (*p* < 0.05). Therefore, the feeding system had a substantial effect on the odor and taste traits of lamb.

### 3.3. Comparison of Fatty Acid Composition between the CF and PF Groups

**Figure 2:**
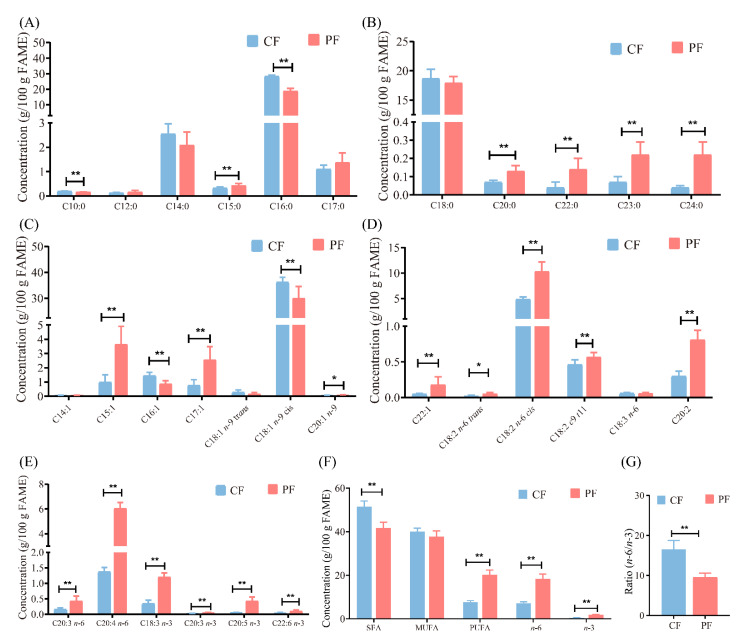
Comparison of fatty acid composition in concentrate-fed (CF) and pasture-fed (PF) groups (*n* = 8). (**A**–**E**) Concentrations of the fatty acids detected. (**F**) Contents of SFA, MUFA, PUFA, *n*-6 and *n*-3 PUFA in lamb. (**G**) The ratio of *n*-6 to *n*-3 PUFA. SFA, saturated fatty acids, MUFA, monounsaturated fatty acids, PUFA, polyunsaturated fatty acids. * *p* < 0.05, ** *p* < 0.01.

Fatty acid composition in the CF and PF groups in this study are displayed in Figure 2. In comparison with the CF group, the amounts of palmitic acid (C16:0) and oleic acid (C18:1 *n*-9 *cis*) markedly reduced in the PF group (*p* < 0.01). Conversely, the PF group had higher levels of linoleic acid (C18:2 *n*-6), eicosatetraenoic acid (C20:5 *n*-3), conjugated linoleic acid (C18:2 *c*9 *t*11), α-linolenic acid (C18:3 *n*-3), and docosahexaenoic acid (C22:6 *n*-3) than the CF group (*p* < 0.01). The amounts of saturated fatty acids markedly decreased in the PF group against the CF group (*p* < 0.01). The contents of *n*-6 and *n*-3 polyunsaturated fatty acids and total polyunsaturated fatty acids were markedly higher in the PF group compared with the CF group (*p* < 0.01). Furthermore, the ratio of *n*-6 to *n*-3 polyunsaturated fatty acids significantly decreased in the PF group (*p* < 0.01). Hence, the feeding system played a critical role in the fatty acid composition of lamb, especially for *n*-3 polyunsaturated fatty acids.

### 3.4. Changes in Hydrophilic Metabolites between the CF and PF Groups

**Figure 3:**
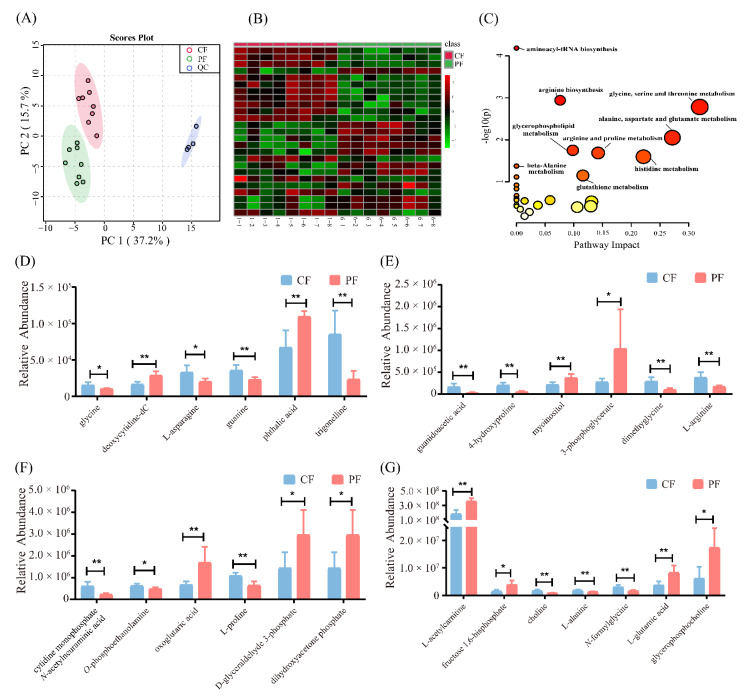
Hydrophilic metabolites in concentrate-fed (CF) and pasture-fed (PF) groups (*n* = 8). (**A**) Score plots of principal component analysis of the identified metabolites. (**B**) Heatmap and (**D**–**G**) bar graphs present the significant metabolites between the CF and PF groups. The significantly increased and decreased levels of metabolites were represented as the red and green cells, respectively, in the heatmap. (**C**) Pathway analysis of significantly different metabolites by means of MetaboAnalyst 5.0 online. QC, quality control samples. * *p* < 0.05, ** *p* < 0.01.

Through a targeted metabolomics approach, we identified a total of 163 different hydrophilic metabolites in longissimus thoracis from Tan sheep (Table S3). The stability of this analytical method was proved by quality control samples presented in Figure 3A, which was satisfactory for use in analyzing hydrophilic metabolites. In order to evaluate the difference of hydrophilic metabolites in the grazing and indoor-fed lambs, the metabolomics data were analyzed with univariate and multivariate statistics. As displayed in Figure 3A, 54.9% of the variation was accounted for the first two principal components, and there was an obvious separation between the CF and PF groups. According to *T*-tests results, 25 hydrophilic metabolites were significantly altered in the CF and PF groups and were visualized with heatmap analysis (false discovery rate < 0.05, Figure 3B). Of these, 11 metabolites, namely, deoxycytidine-dC, phthalic acid, myoinositol, 3-phosphoglycerate, oxoglutaric acid, d-glyceraldehyde 3-phosphate, dihydroxyacetone phosphate, l-acetylcarnitine, fructose 1,6-bisphosphate, l-glutamic acid, and glycerophosphocholine, significantly increased in longissimus thoracis from the PF group compared with the CF group. Additionally, 14 metabolites (glycine, l-asparagine, guanine, trigonelline, guanidoacetic acid, 4-hydroxyproline, dimethyglycine, l-arginine, cytidine monophosphate *N*-acetylneuraminic acid, *O*-phosphoethanolamine, l-proline, choline, l-alanine, and *N*-formylglycine) remarkably decreased in the PF group (Figure 3D–G). Pathway analysis was performed with 25 significantly different metabolites by Metaboanalyst 5.0 online. Eight pathways with *p* < 0.05 were identified: aminoacyl-tRNA biosynthesis; arginine biosynthesis; glycine, serine, and threonine metabolism; glycerophospholipid metabolism; histidine metabolism; arginine and proline metabolism; alanine, aspartate, and glutamate metabolism; and β-alanine metabolism (Figure 3C).

### 3.5. Comparison of Volatile Compounds between the CF and PF Groups

**Figure 4:**
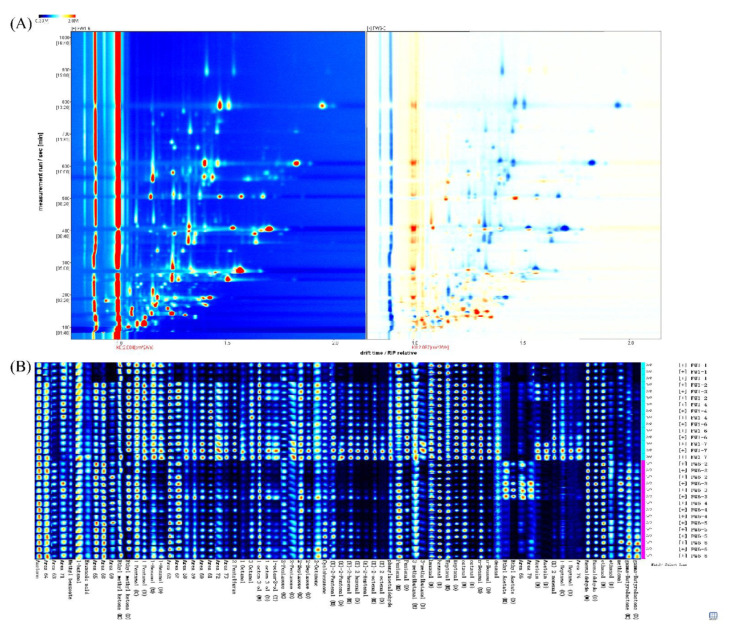
Volatile compound profiles in concentrate-fed (CF) and pasture-fed (PF) groups (*n* = 5). (**A**) Two-dimensional topographic plots for lamb with different feeding systems. The darker color indicates greater intensity (red represents to high levels of volatiles and white represents to low levels). (**B**) Gallery plot of the selected signal intensities collected with different feeding systems.

Volatile compounds of the grazing and indoor-fed lambs were investigated using GC–IMS, and the results are displayed as the topographic plot in Figure 4A. Volatile compounds of lambs from different feeding systems appeared in the retention time of 100–800 s and with a 1.0–1.5 ms drift time. We presented similar visualizations of the CF and PF groups, but the signal intensity differed between the two groups. This result suggests that the volatile profiles of the CF and PF groups were different.

**Figure 5:**
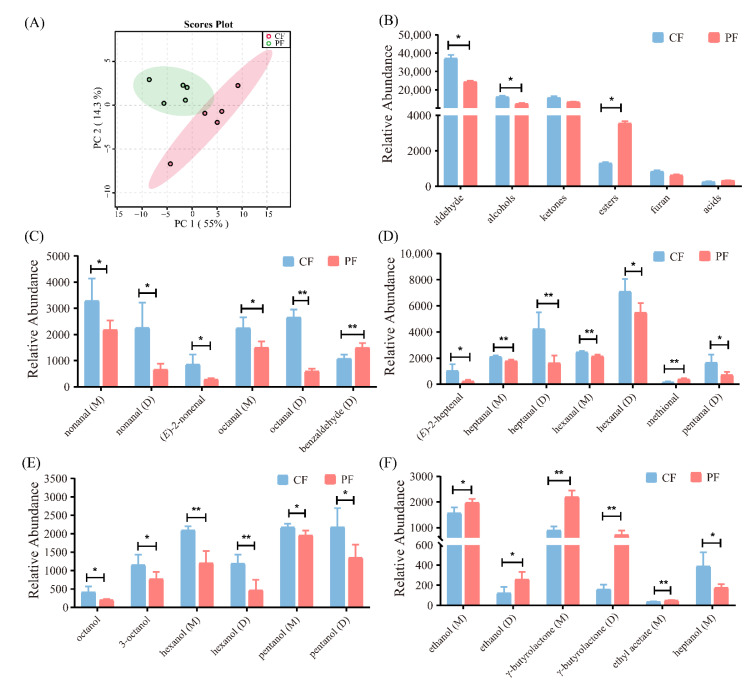
Comparison of the identified volatile compounds in concentrate-fed (CF) and pasture-fed (PF) groups (*n* = 5). (**A**) Principal component analysis score plots of volatile compounds in lamb with different feeding systems. (**B**) Comparison of volatile categories identified in the CF and PF groups. (**C**–**F**) Bar graphs present the significant volatile compounds between the CF and PF groups. M, monomer; D, dimer. * *p* < 0.05, ** *p* < 0.01.

Volatile compound fingerprints of the CF and PF groups were revealed in a gallery plot, and the different volatile compounds in lambs with different feeding systems were clearly observed. A total of 57 volatile compounds were identified, namely, 23 aldehydes, 16 alcohols, 11 ketones, 5 esters, 1 furan, and 1 acid (Figure 4B and Table S4). Due to different concentrations, it was observed that some compounds showed multiple signals (monomer (M) and dimer (D), or even trimer). Monomers such as hexanal, pentanal, and 1-octen-3-ol were eluted before dimers and trimers, because of their lower mass. Figure 5A showed an evident separation between the CF and PF samples. In comparison with the CF group, the levels of esters were significantly higher while the concentrations of aldehydes and alcohols were remarkably lower in the PF group (*p* < 0.05, Figure 5B). Additionally, *T*-tests were performed to evaluate the difference in volatile compounds between the CF and PF group, and the contents of 25 volatile compounds in lamb following different feeding systems were changed (Figure 5C–F). Of these, the levels of methional, ethanol (M), ethanol (D), γ-butyrolactone (M), γ-butyrolactone (D), and ethyl acetate (M) significantly raised in the PF group against the CF group. However, a significant decline was observed in the contents of 19 volatile compounds, namely, nonanal (M), nonanal (D), (*E*)-2-heptenal, octanol, 3-octanol, (*E*)-2-nonenal, octanal (M), octanal (D), heptanal (M), heptanal (D), benzaldehyde (D), pentanal (D), hexanal (M), hexanal (D), hexanol (M), hexanol (D), pentanol (M), pentanol (D), and heptanol (M) in the PF group. Collectively, these significantly altered volatile compounds might be responsible for the difference in aroma profiles of the CF and PF groups.

## 4. Discussion

Grazing is a traditional feeding system and greatly contributes to lamb flavor. Pastoral odor is a characteristic odor of lambs fed with pasture, which consumers greatly prefer in China and Australia because of their diet cultures. In this study, the PF group had higher pH_45 min_, pH_24 h_, a*_45 min_, a*_24 h_, and shear force values, as well as lower IMF content in comparison with the CF group. Similarly, previous studies noted that the grazing lamb showed redder color and higher pH_24h_ values relative to the indoor-fed lamb [23,24]. The great reduction of IMF content may influence some sensory attributes, leading to different consumer attitudes toward grazing and indoor-fed lambs.

This distinctiveness of meat quality traits and IMF content could be associated with high levels of exercise and forage in Tan sheep fed by pasture. High levels of exercise led to low accumulation of muscle glycogen and low glycolytic potential [25,26]. Moreover, the high energy expenditures led to inefficient deposition and a high usage of lipids, thus lowering the IMF content [23,27,28,29,30]. High physical activity of grazing sheep could result in high myoglobin content, as well as increased diameters of muscle fibers and a degree of myofibrillar protein cross-linking, affecting meat color attributes and shear force [31]. Additionally, fresh foraged plants are rich in many antioxidant ingredients (i.e., selenium and vitamin E) and may increase the antioxidant activity, improving meat color stability [32].

Based on the results obtained from the fatty acid analysis, grazing greatly altered the fatty acid composition. Levels of palmitic acid decreased by 33.33% in the PF group, which was consistent with previous reports [33]. The change may be accounted for by the fact that the high dietary energy from concentrate increases because of de novo synthesis of C12–C16 [34]. It is reported that the proportion of palmitic acid increases with an increasing quantity of concentrates in the diet [33]. The contents of oleic acid in the PF group significantly decreased by 17.18% to 30.04%. This reduction in oleic acid may be related to dietary composition. It is noted that high oleic acid in a cereal diet could promote the accumulation of oleic acid in muscle. Moreover, the high IMF content in the CF group may increase the activity of ∆9 desaturase enzymes, increasing oleic acid content [35]. The PF group contained 1.13 times more linoleic acid than the CF group, consistent with previous reports [30,31]. It is found that IMF content was negatively correlated with linoleic acid level in longissimus thoracis, and that the proportion of linoleic acid in phospholipid decreased with the declining proportion of phospholipid in IMF as IMF content increased [36]. The level of α-linolenic acid in the PF group significantly increased by 2.5 times—up to 1.20% in comparison with the CF group, consistent with previously reported results [37]. Some of the α-linolenic acid escapes ruminal biohydrogenation and elongates to *n*-3 long-chain polyunsaturated fatty acids (i.e., eicosapentaenoic acid), which are beneficial to human growth and maintenance of cognitive function [27]. Additionally, the *n*-6/*n*-3 ratio was remarkably lower in the PF group against the CF group, similar to the result reported [38]. The lower *n*-6/*n*-3 ratio can prevent or modulate some human diseases and meet consumers’ demand for meat. Therefore, grazing may regulate the fatty acid profiles of Tan sheep, especially for *n*-3 polyunsaturated fatty acids, by dietary composition and some enzyme activity.

The most of volatile compounds detected in the grazing and indoor-fed lamb have been reported [10,39]. The predominant volatile compounds in this study were aldehydes with low odor thresholds. The CF group had 38.70% more benzaldehyde than the CF group, in line with previous reports [40]. Benzaldehyde has a bitter almond note, which could be formed by linolenic acid degradation [7] and has been evidenced in cooked meat [22]. The levels of methional were approximately 1.21 times higher in the PF group versus the CF group. Methional was the only sulfur-containing compound detected in lambs under different feeding regimes which possesses a cooked potato odor and originates from the Strecker degradation reaction between methionine and glucose. It has been evidenced that the difference in methional is related to diet differences between concentrate and pasture [6]. γ-Butyrolactone (M) in the PF group, which was previously proposed as a pasture-diet tracer and was found to be conducive for desirable sweet and fatty flavors, significantly increased by 146.44% [41]. Additionally, the PF samples had less lipid-derived compounds, including saturated aldehydes, saturated alcohols, (*E*)-2-nonenal, and (*E*)-2-heptenal, as compared with the CF samples. Similarly, it was noted that the grazing lambs had low concentrations of lipid-generated alkenals, including (*E*)-2-heptenal, (*E*)-2-octenal, and (*E*)-2-nonenal [6]. The concentrations of nonanal (M) and octanal (M) significantly decreased by 33.62% and 33.55%, respectively, in the PF group. Nonanal and octanal have a fatty and fruity odor, and can be generated by the thermal oxidation and autoxidation of *n*-9 monounsaturated fatty acids (i.e., oleic acid) [42]. The difference in volatile compounds between the CF and PF groups may be due to the protection of antioxidants existing in the diet naturally [6] and the high contents of *n*-3 polyunsaturated fatty acids. It has been reported that an increase in *n*-3 polyunsaturated fatty acids may affect the flavor formation by influencing the interaction between the lipid-derived compounds and the Maillard reaction compounds, and by promoting the oxidation of *n*-6 and *n*-9 fatty acids [8]. Therefore, grazing mainly contributes to the concentration of volatile compounds in lamb, thus altering the lamb’s aroma profile.

## 5. Conclusions

In summary, we investigated the influence of feeding systems on meat quality traits, fatty acid profiles, hydrophilic metabolites, and volatile compound profiles in Tan sheep by using foodomics analysis. The muscles from lamb finished on pasture exhibited higher pH_24h_ value, redder color, and lower IMF content. E-nose and E-tongue analyses indicated that the taste and odor profiles of the PF lambs were different from those of the CF lambs. Metabolomics showed that 25 of the 163 hydrophilic metabolites identified, which were involved in amino acid and glycolipid metabolism, were significantly affected by the feeding system. Volatile compound profiles of the CF and PF groups significantly differed. The levels of methional, γ-butyrolactone, benzaldehyde, and ethyl acetate were markedly higher in the PF group against the CF group, whereas the lipid-derived saturated aldehydes, alcohols, and alkenals were remarkably lower in the PF group. Our findings provide an initial view of the influence of grazing on flavor precursors and volatilomic profiles in lamb, which may be beneficial to improving the meat quality of indoor-fed Tan sheep.

## Figures and Tables

**Table 1:** Meat quality characteristics and intramuscular fat content in concentrate-fed (CF) and pasture-fed (PF) groups (*n* = 14).

Item	CF	PF	*p*-Value
pH_45 min_	6.54 ± 0.22	6.71 ± 0.18	0.038
pH_24 h_	5.79 ± 0.12	5.89 ± 0.08	0.023
L*_45 min_	36.42 ± 2.61	36.74 ± 2.00	0.718
a*_45 min_	17.93 ± 2.51	20.27 ± 1.98	0.011
b*_45 min_	6.20 ± 1.43	7.00 ± 0.90	0.088
L*_24 h_	39.70 ± 2.62	45.04 ± 2.10	<0.01
a*_24 h_	18.77 ± 1.40	22.06 ± 1.64	<0.01
b*_24 h_	9.15 ± 1.48	8.64 ± 0.81	0.215
Drip loss (%)	2.50 ± 0.47	2.24 ± 0.22	0.085
Cooking loss (%)	31.18 ± 2.26	32.60 ± 4.40	0.461
Shear force (*n*)	63.81 ± 11.61	82.02 ± 4.64	<0.01
Texture profile analysis			
Hardness (g)	9605.81 ± 639.48	11,437.96 ± 1082.00	<0.01
Chewiness (g)	2755.01 ± 553.16	3892.81 ± 944.91	0.016
Gumminess (g)	5530.03 ± 498.46	6208.93 ± 963.46	0.151
Cohesiveness (%)	58.24 ± 1.20	64.40 ± 3.18	<0.01
Springiness (%)	53.94 ± 3.19	49.77 ± 4.07	0.071
Intramuscular fat content (%)	3.96 ± 1.06	1.50 ± 0.31	<0.01

## Data Availability

The data presented in this study are available in the Supplementary Material.

## Supplementary Materials

The following supporting information can be downloaded at: https://www.mdpi.com/article/10.3390/foods11020191/s1, Table S1: The basic diet composition and nutritional level of the concentrate-fed group (dry matter basis), Table S2: Performance description and sensitivity of metal oxide sensors of the PEN3 E-nose, Table S3: Hydrophilic metabolites identified in concentrate- and pasture-fed groups using targeted metabolomics, Table S4: Volatile compounds identified in concentrate-fed (CF) and pasture-fed (PF) groups by using GC–IMS-based volatilomics.
